# Evolution, gene expression profiling and 3D modeling of CSLD proteins in cotton

**DOI:** 10.1186/s12870-017-1063-x

**Published:** 2017-07-10

**Authors:** Yanpeng Li, Tiegang Yang, Dandan Dai, Ying Hu, Xiaoyang Guo, Hongxia Guo

**Affiliations:** 1 Industrial Crop Research Institute, Henan Academy of Agricultural Sciences, No. 116, Huayuan Road, Zhengzhou, 450002 China; 2 Scientific Observing and Experimental Station of Crop Cultivation in Central Plain, Ministry of Agriculture, No. 116, Huayuan Road, Zhengzhou, 450002 China

**Keywords:** Cotton, CSLD, Phylogenetic tree, Positive selection, *CSL* superfamily, Structural modeling, Cellulose synthase, Cell wall

## Abstract

**Background:**

Among CESA-like gene superfamily, the cellulose synthase-like D (*CSLD*) genes are most similar to cellulose synthase genes and have been reported to be involved in tip-growing cell and stem development. However, there has been no genome-wide characterization of this gene subfamily in cotton. We thus sought to analyze the evolution and functional characterization of CSLD proteins in cotton based on fully sequenced cotton genomes.

**Results:**

A total of 23 full-length CSLD proteins were identified in *Gossypium raimondii*, *Gossypium arboreum* and *Gossypium hirsutum.* The phylogenetic tree divided the CSLD proteins into five clades with strong support: CSLD1, CSLD2/3, CSLD4, CSLD5 and CSLD6. The total expression of *GhCSLD* genes was the highest in androecium & gynoecium (mostly contributed by *CSLD1* and *CSLD4*) compared with other *CSL* genes. *CSLD1* and *CSLD4* were only highly expressed in androecium & gynoecium (A&G), and showed tissue-specific expression. The total expression of *CSLD2/3, 5 and 6* was highest in the specific tissues. These results suggest that *CSLD* genes showed the different pattern of expression. Cotton CSLD proteins were subjected to different evolutionary pressures, and the CSLD1 and CSLD4 proteins exhibited episodic and long-term shift positive selection. The predicted three-dimensional structure of GrCSLD1 suggested that GrCSLD1 belongs to glycosyltransferase family 2. The amino acid residues under positive selection in the CSLD1 lineage are positioned in a region adjacent to the class-specific region (CSR), β1-strand and transmembrane helices (TMHs) in the GrCSLD1structure.

**Conclusion:**

Our results characterized the CSLD proteins by an integrated approach containing phylogeny, transcriptional profiling and 3D modeling. The study added to the understanding about the importance of the CSLD family and provide a useful reference for selecting candidate genes and their associations with the biosynthesis of the cell wall in cotton.

**Electronic supplementary material:**

The online version of this article (doi:10.1186/s12870-017-1063-x) contains supplementary material, which is available to authorized users.

## Background

The plant cell wall plays a central role in plant development and is primarily composed of three polysaccharides: cellulose, hemicellulose and pectin [1–3]. Lignin is a major polymer of secondary cell wall [4]. Cellulose comprises unbranched homopolymers of β-1,4-linked glucose units and is a core structural component of the plant cell wall [5]. The biosynthesis of cellulose has attracted great interest because cellulose microfibrils are key determinants of the physical characteristics of the cell wall [6]; provide renewable resources for biofuels [7, 8]. Cellulose is synthesized by cellulose synthase (CESA) which belongs to glycosyltransferase family 2 (GT2) [9]. The CSLs, which are grouped into 10 families (CSLA, B, C, D, E, F, G, H, J, and K), and CESA form the CESA superfamily [10–12]. However, there are six families (CSLA, C, D, E, F and H) in rice [13, 14]. The CSLs are also members of GT2 [9]. In plants, *CESA* genes were first identified in cotton fiber based on sequence homology to bacterial *CESA* genes [15]. The nearly complete genome sequence of the *Arabidopsis thaliana* revealed 10 *CESA* genes [2, 12], which are classified as required for primary (*CESA1*, *2*, *3*, *5*, *6*, *9*) and secondary (*CESA4*, *7*, *8*) cell wall synthesis [16–18]. CESA1, CESA3 and CESA6 are considered parts of the primary wall CESA complex, and CESA5 and CESA2 are partially functionally redundant with CESA6 at different stages of growth [16]. CESA6-related CESA9 exhibits functional redundancy with CESA6 [17]. The secondary wall CESA complex comprises CESA4, CESA7 and CESA8, as identified in *irx* (*irregular xylem*) mutants of *A. thaliana* [18]. In contrast to the primary wall CESA complex, these three *CESA* genes appear equally important for cellulose synthesis in the secondary cell wall, indicating that they are not redundant with one another [18]. In cotton, CESA1, 2, 7, 8 (the orthologs of *A. thaliana* CESA8, 4, 7 and 7, respectively) are associated in the cellulose biosynthesis secondary cell wall, whereas CESA3, 5, 6, 9 and 10 (the orthologs of *A. thaliana* CESA3, 2/5/6/9, 1/10, 2/5/6/9 and 3, respectively) participate in primary cell wall synthesis in cotton fiber [19–22]. Moreover, CESA8 (ATCESA7) could paly an enhancer role for rapid and massive cellulose accumulation of secondary cell wall in cotton fiber development, which is quite different from other grass species [19]. More recently, it has been reported that there is a “relay race” model for fiber development involving the *CesA* genes in *G. barbadense* [23].

*CSL* genes encoding processive glycosyltransferases have been indicated in the biosynthesis of non-cellulosic polysaccharides in the plant wall. For instance, *CSLA* genes encode mannan synthases [24, 25], *CSLC* genes encode β-1,4 glucan synthases that mediate xyloglucan biosynthesis [26], and the CSLF and CSLH proteins are involved in (1,3;1,4)-β-D-glucan biosynthesis [27, 28]. Among *CSL* gene families, the *CSLD* gene family is most similar to the CESA family and possesses the most ancient intron/exon structure [12]. The CSLDs have been implicated in cellulose and mannan synthesis [29–31]. In *A. thaliana*, mutants in five *CSLD* genes have been described to cause distinct phenotypes. In *CSLD3* mutants, root hairs form bulges soon after initiation [32, 33], and CSLD3 is involved in the synthesis of β-1,4-glucan polysaccharide in the apical plasma membrane of root hair cells [29]. *CSLD2* mutants grow abnormal root hairs [34], and there may be partial divergence and redundancy in *CSLD2* and *CSLD3* gene function during root hair and female gametophyte development [34, 35]. *CSLD5* mutants have significantly reduced stem and root growth [36]. A recent report showed that CSLD5 participates in the construction of newly forming cell plates and is an unstable protein that is degraded upon completion of cell division [37]. Furthermore, the cooperative activities of *CSLD2*, *CSLD3* and *CSLD5* are necessary for normal development [31]. *CSLD1* and *CSLD4* mutants exhibit a significant reduction of cellulose deposition on pollen tubes and distinct disorder of pollen tube wall layers, suggesting that the *CSLD1* and *CSLD4* genes are required for normal pollen tube growth [30, 34]. In rice, mutations in *OsCSLD1* and *OsCSLD4*, the orthologs of *A. thaliana CSLD2/3* and *CSLD5*, respectively, have an important influence on leaf morphogenesis and plant architecture [38–41]. *OsCSLD1* mutants exhibit abnormal root hair [38]. Maize CSLD1 (the ortholog of *A. thaliana* CSLD5) is required for cell division, expansion and leaf growth [42]. The major studies mentioned above indicate that CSLD proteins may be involved in cellulose synthesis in tip-growing cells (pollen tubes and root hairs) and stem growth.

Cotton is one of the most economically important crops, and its fiber is the main natural source for the textile industry [43]. Cotton is also an excellent model system for the study of polyploidization, cell wall biosynthesis and cell elongation [44–46]. Despite outstanding progress in *A. thaliana* and rice, little is known regarding CSLD proteins in cotton. The cotton *CSL* genes are involved in the synthesis of cell wall matrix polysaccharides that surround cellulose microfibrils in cotton [20]. The genes *CSLD2/3* and *CSLD6* but not *CSLD1* and *CSLD4* are expressed strongly in fiber development [21, 45]. The *CSLD2/3* genes have also been suggested to be involved in mannan synthesis during cotton fiber cell development [47].

The recently assembled and published genome sequences for *Gossypium raimondii* [20], *Gossypium arboretum* [48] and *Gossypium hirsutum* [45] provide an opportunity to identify and analyze the *CSL* gene family at the whole-genome level. Here, to gain insight into the evolution and functional characterization of CSLD proteins based on the cotton genome, we identified the CSLD proteins and constructed maximum likelihood (ML) and Bayesian phylogenetic trees to reconstruct the evolutionary origin of the *CSLD* genes. Then, gene expression, qRT-PCR and positive selection were analyzed. Finally, we generated a model of the three-dimensional structure of CSLD1 to elucidate the function of CSLD1. We show that 1) the 23 full-length CSLD proteins are divided into five clades; 2) *CSLD* genes show the different expression patterns compared with CESA and other *CSL* genes; 3) the CSLD1 and CSLD4 clades exhibit episodic and long-term shift positive selection; 4) the GrCSLD1 protein belongs to glycosyltransferase family 2 and probably participates in the biosynthesis of cellulose, mannan or other polysaccharides. These results provide a thorough picture of the evolution and biological and molecular function of CSLD proteins in cotton.

## Results

### Distribution of CSLD proteins in the cotton genome

**Fig. 1:**
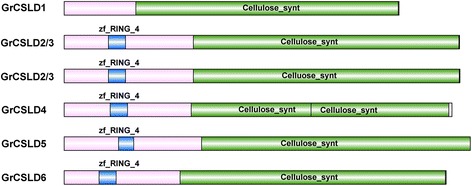
The domain architecture illustrated using IBS software [112]. GrCSLD, CSLD protein in *G. raimondii* according to the phylogenetic tree of CSLD proteins in Fig. 3; zf-RING_4, zf-RING domain (blue); cellulose_synt, cellulose_synt domain (green). GrCSLD2/3, GrCSLD5 and GrCSLD6 have a cellulose_synt and a zf-RING_4 domain. GrCSLD1 only contains a cellulose_synt domain, and GrCSLD4 includes a zf-RING_4 and two cellulose_synt domains. Two GrCSLD2/3 s are shown in Fig. 1 because Gorai.004G257300.1, Gorai.003G052200.1, AT_CSLD2 and AT_CSLD3 form a monophyletic group (Fig. 3). The functional domains and positions of these domains were identified via sequence searches with the online programs SMART, Interpro and NCBI conserved domain databases

**Fig. 2:**
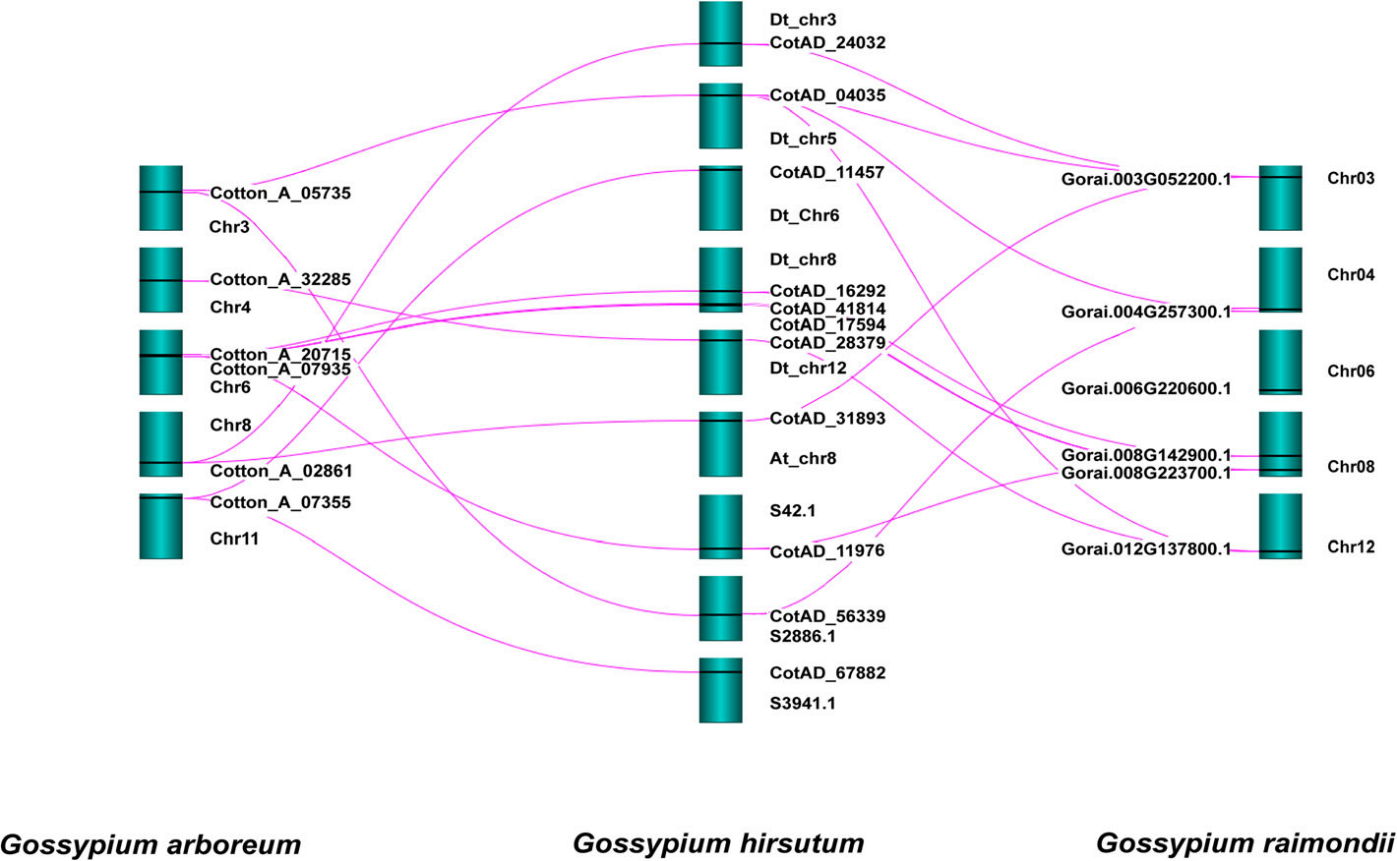
Conserved syntenic positions in *G. arboreum* and *G. raimondii* compared with *G. hirsutum* using Strudel software. The pink lines show syntenic relationships between *G. hirsutum* and *G. arboreum* or *G. raimondii*. The positions of the *CSLD* genes on the respective chromosomes (dark cyan) are indicated using black lines

The availability of complete genome sequences from cotton provides an opportunity to identify and analyze the evolution and function of the CSLD proteins. 1923 Mb (88.5%), 1532 Mb (90.4%) and 761.4 Mb (99.95%) is anchored and oriented to 26 pseudochromosomes in *G. hirsutum* [45], to 13 pseudochromosomes in *G. arboretum* [48] and to 13 pseudochromosomes *G. raimondii* [20], respectively. Based on a homology-based protein search using confirmed functional CSLD proteins, we identified 23 full-length CSLD proteins from *G. arboretum* (six), *G. hirsutum* (11) and *G. raimondii* (six) (Table 1) and 86 CSLD proteins from 15 other plant species, as expected (Additional file 1: Table S1, Additional file 2). *G. hirsutum* has approximately twice as many CLSD proteins as *G. arboreum* or *G. raimondii*. In cotton, some CSLD proteins have one or two cellulose_synt (PF03552) domains and a zf-RING_4 (PF14570) domain, but the others have only one or two cellulose_synt (PF03552) domains (Fig. 1). The *CSLD* genes are distributed on six chromosomes (Dt_chr3, 5, 6, 8, 12 and At_chr8) and three scaffolds (S42.1, S2886.1, and S3941.1) in *G. hirsutum*, five chromosomes (Chr3, 4, 6, 8, and 11) in *G. arboreum*, and five chromosomes (Chr03, 04, 06, 08, and 12) in *G. raimondii*. Most chromosomes distributing the *CSLD* genes contain a single locus *of CSLD* genes, except chromosome 6 in *G. arboreum*, chromosome Dt_Chr8 in *G. hirsutum* and chromosome 08 in *G. raimondii*, which contain 2, 3 and 2 *CSLD* gene loci, respectively (Fig. 2). The syntenic positions for *G. arboreum* and *G. raimondii* were compared with those of *G. hirsutum* (Fig. 2). One-to-two syntenic relationships were identified between *G. arboreum* or *G. raimondii* and *G. hirsutum* except for Chr4 (one-to-one syntenic relationship between *G. arboreum and G. hirsutum*), Chr06 (no syntenic relationship between *G. raimondii and G. hirsutum*) and Chr03 (one-to-three syntenic relationships between *G. raimondii and G. hirsutum*).

**Table 1:** Chromosomal locus ID and length of CSLD proteins in cotton

Organism	CSLD protein	Gene name^a^	Exon	Locus ID	Strand	Length
*G.arboreum*	Cotton_A_07355	*GaCSLD1*	3	Chr11:7526483-7529728	+	1006
	Cotton_A_05735	*GaCSLD2/3*	2	Chr3:41008,691-41012256	−	1160
	Cotton_A_02861	*GaCSLD2/3*	3	Chr8:99437456-99441339	+	1144
	Cotton_A_32285	*GaCSLD4*	5	Chr4:70165526-70169404	−	1144
	Cotton_A_07935	*GaCSLD6*	3	Chr6:50169413-50173209	−	1104
	Cotton_A_20715	*GaCSLD5*	3	Chr6:48564230-48568215	+	1175
*G.hirsutum*	CotAD_11457	*GhCSLD1*	3	Dt_Chr6:3724390-3727635	−	1006
	CotAD_67882	*GhCSLD1*	3	Scaffold3941.1:14327-17572	−	1006
	CotAD_04035	*GhCSLD2/3*	2	Dt_chr5:12149740-12153305	+	1143
	CotAD_56339	*GhCSLD2/3*	2	Scaffold2886.1:98206-101771	+	1143
	CotAD_31893	*GhCSLD2/3*	3	At_chr8:11464660-11468543	+	1144
	CotAD_24032	*GhCSLD2/3*	3	Dt_chr3:27558271-27562152	+	1144
	CotAD_28379	*GhCSLD4*	4	Dt_chr12:6056272-6060210	−	1121
	CotAD_17594	*GhCSLD6*	3	Dt_chr8:49809841:49813637	+	1104
	CotAD_16292	*GhCSLD5*	3	Dt_chr8:38197719-38201702	−	1175
	CotAD_41814	*GhCSLD6*	3	Dt_chr8:48706763-48710552	−	1104
	CotAD_11976	*GhCSLD5*	3	Scaffold42.1:2430018-2434003	+	1175
*G.raimondii*	Gorai.006G220600.1	*GrCSLD1*	3	Chr06:47301257-47304509	+	968
	Gorai.004G257300.1	*GrCSLD2/3* ^b^	3	Chr04:59350347-59355312	+	1143
	Gorai.003G052200.1	*GrCSLD2/3* ^b^	4	Chr03:7953077-7958739	−	1144
	Gorai.012G137800.1	*GrCSLD4*	4	Chr12:31115815-31119908	−	1121
	Gorai.008G142900.1	*GrCSLD5*	3	Chr08:39398378-39402804	−	1174
	Gorai.008G223700.1	*GrCSLD6*	4	Chr08:51011238-51015956	−	1104

^a^Gene names refer to the phylogenetic tree of CSLD proteins in Fig. 3

^b^ *CSLD2* and *CSLD3* are designated *CSLD2/3* because of two closely related isoforms in *A. thaliana*

### Evolution of cotton CSLD proteins

To reconstruct the phylogenetic trees, we used different alignment methods, evolutionary models, and multiple statistical-support measures (see the Methods section for details). Each alignment was analyzed with ProtTest3.2 to select the most appropriate amino acid substitution model for inferred maximum likelihood (ML) phylogenetic trees. The LG + I + G + F model was chosen as the best model according to AIC, AICc and BIC criteria (Additional file 3: Table S2). To further verify the robustness of the phylogenetic trees reconstructed by PhyML, we inferred Bayesian phylogenetic trees under a mixed model using MrBayes, which integrated over all available substitution models instead of specifying an amino acid substitution model [49]. In this approach, each of the multiple amino acid substitution models contributes to the result in proportion to its posterior probability. A comparison of phylogenetic trees obtained from ML and Bayesian methods using Ktreedist is shown in Additional file 4: Table S3. Based on the K-scores and symmetric differences (Robinson-Foulds distance), the ML and Bayesian trees based on elision and Muscle alignments exhibited nearly identical topology and branch lengths (K-score, 0.09 and 0.13; symmetric difference, 4 and 5). However, the ML and Bayesian trees based on two alignments (Kalign and Mafft) exhibited greater topological differences than the elision and Muscle alignments.

**Fig. 3:**
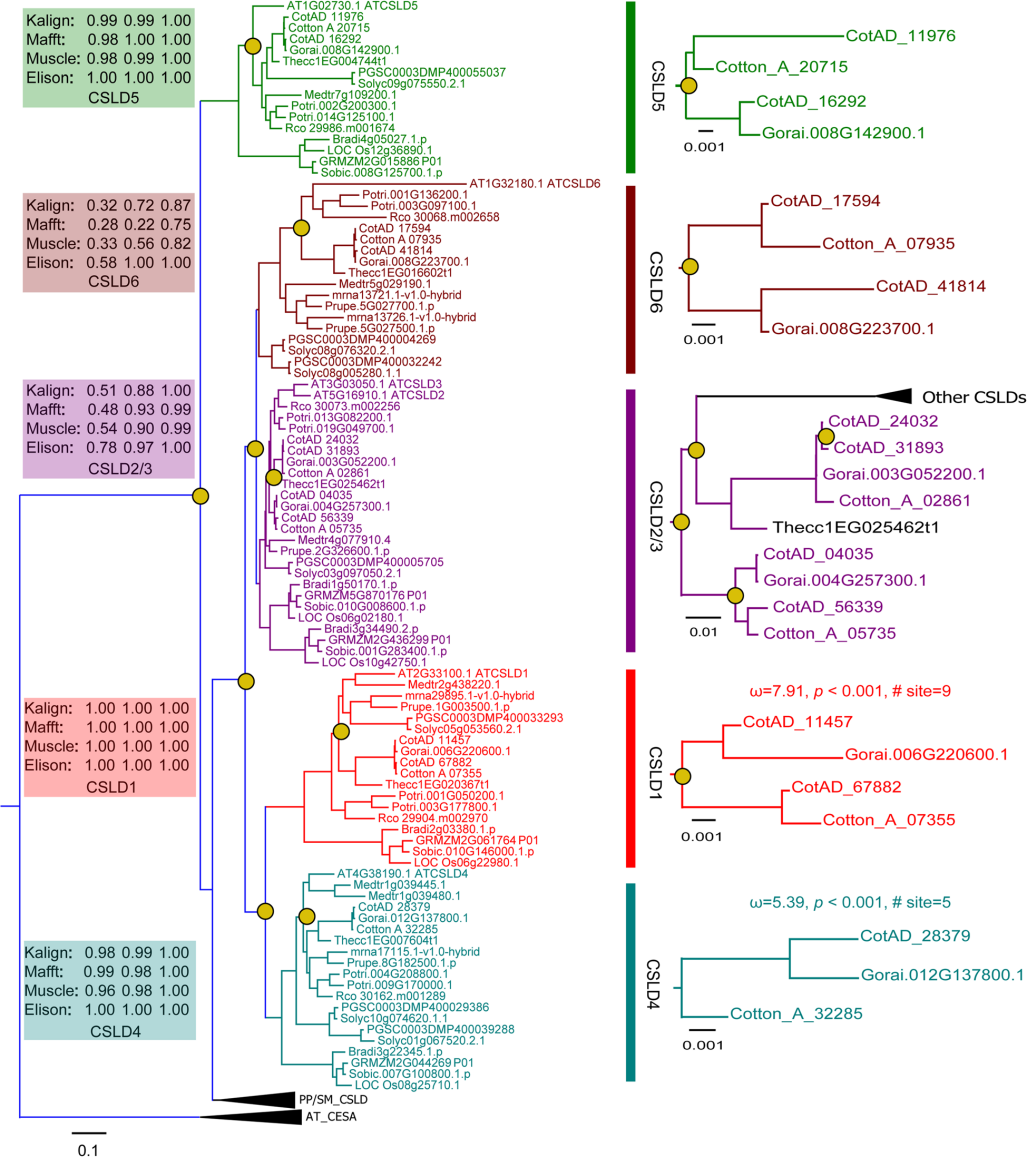
Phylogenetic analysis of the CSLD proteins in *Gossypium* and 15 other plant species using *A. thaliana* CESA genes as an outgroup (Additional file 1: Table S1). The phylogenetic tree was inferred using maximum likelihood and Bayesian methods. Support values are shown for key nodes as bootstrap proportions/SH-like aLRT scores/Bayesian posterior probabilities. The CSLD protein clades are indicated in different colors. The duplication events are annotated as brass circles. PP/SM_CSLD indicates CSLD proteins in *P. patens* and *S. moellendorffii*. AT_CESA presents *A. thaliana* CESA proteins as an outgroup. ω denotes dN:dS values. The *P* values were corrected with Bonferroni correction

The Bayesian tree based on elision alignments divided the CSLD proteins into five strongly supported clades: CSLD1, CSLD2/3, CSLD4, CSLD5 and CSLD6, as observed in the model plant *A. thaliana* [12, 35]. Support for the key nodes increased when we used the elision strategy, which concatenates the multiple alignments, and the mixed model method of MrBayes, suggesting that the most reliable alignment positions consistently support a phylogeny in which the CSLD proteins are classed into five clades (Fig. 3). As with the analysis of the whole CSLD phylogenetic tree, the cotton CSLD phylogeny was robustly divided into five clades, and the support values of almost all nodes also increased when we used the elision strategy (Additional file 5: Figure S1). The topological differences based on the three alignments (Kalign, Mafft and Muscle) between the cotton CSLD trees inferred from ML and Bayesian methods are shown in Additional file 5: Figure S1.

The topology of this phylogenetic tree shows that *CSLD* ancestral gene duplication occurred before the moss lineage diverged from vascular plants. Each of the two copies of the ancestral gene evolved separately, leading to the *CSLD5* clade and the other *CSLD* clades (Fig. 3). The topology of the cotton *CSLD* tree is identical to that of the whole *CSLD* phylogenetic tree, which indicates that the cotton *CSLD* ancestor gene split into the *CSLD5* clade and other *CSLD* clades, which were later divided into the *CSLD1*, *CSLD2/3*, *CSLD4*, and *CSLD6* subclades via gene duplication. The CSLD proteins from the three *Gossypium* species form five monophyletic groups, each consisting of three or four CSLD proteins. *CSLD1* and *CSLD2/3* form sister groups to *CSLD4* and *CSLD6*, respectively. The *GhCSLD* genes, except for *CSLD4*, duplicated once again recently (Fig. 3) through hybridization of the two ancestral species approximately 1.5 million years ago (MYA) [45]. Both *G. raimondii* and *G. arboreum* experienced an ancient hexaploidization event that is shared among the eudicots at 115-146 MYA and then underwent a cotton-specific whole genome duplication at 13-20 MYA [43, 48]. These conclusions support the presence of multiple *CSLD* gene copies in the three cotton species.

### Expression profiles of cotton *CSLD* genes

**Fig. 4:**
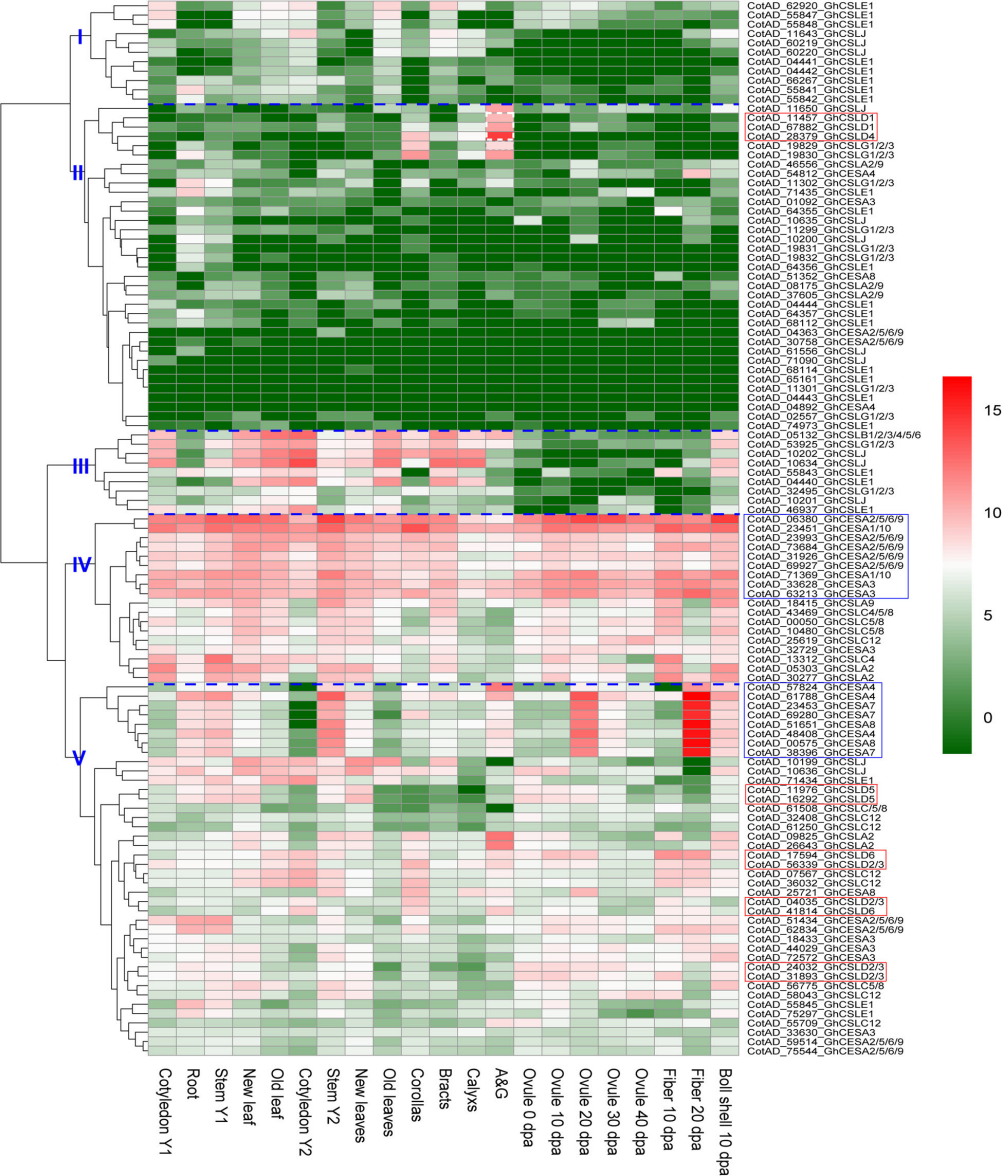
Expression profiling of CESA/CSL genes in *G. hirsutum*. The color key representing the count data that were subjected to variance stabilization transformation in the DESeq packages is shown right. Red, white and green refers to high expression, medium expression and low expression, respectively. I, II, III, IV and V denote five major groups based on the hierarchical clustering analysis using pheatmap

**Fig. 5:**
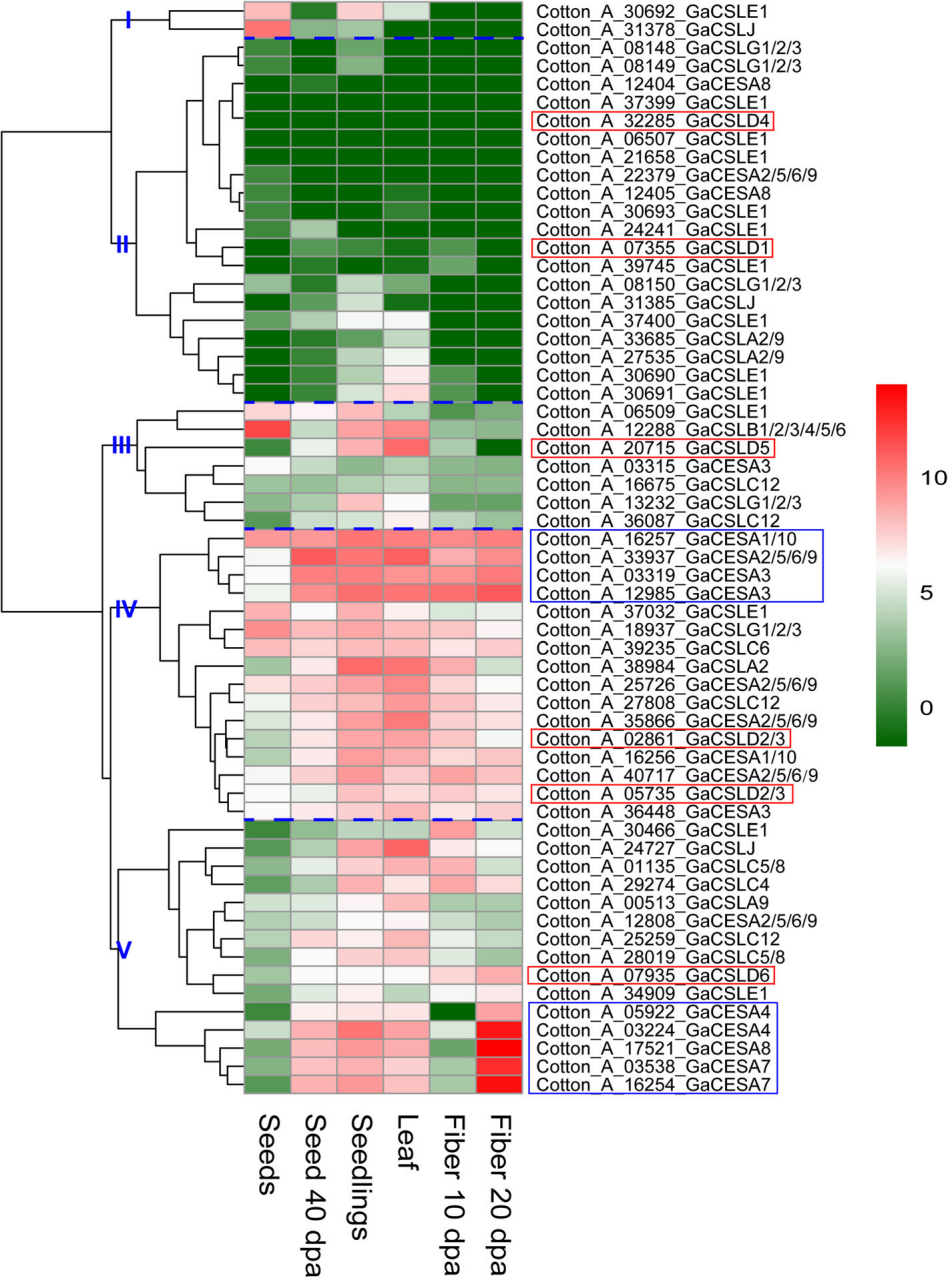
Expression profiling of CESA/CSL gene in *G. arboreum*. The color key representing the count data that were subjected to variance stabilization transformation in the DESeq packages is shown right. Red, white and green refers to high expression, medium expression and low expression, respectively. I, II, III, IV and V denote five major groups based on the hierarchical clustering analysis using pheatmap

**Fig. 6:**
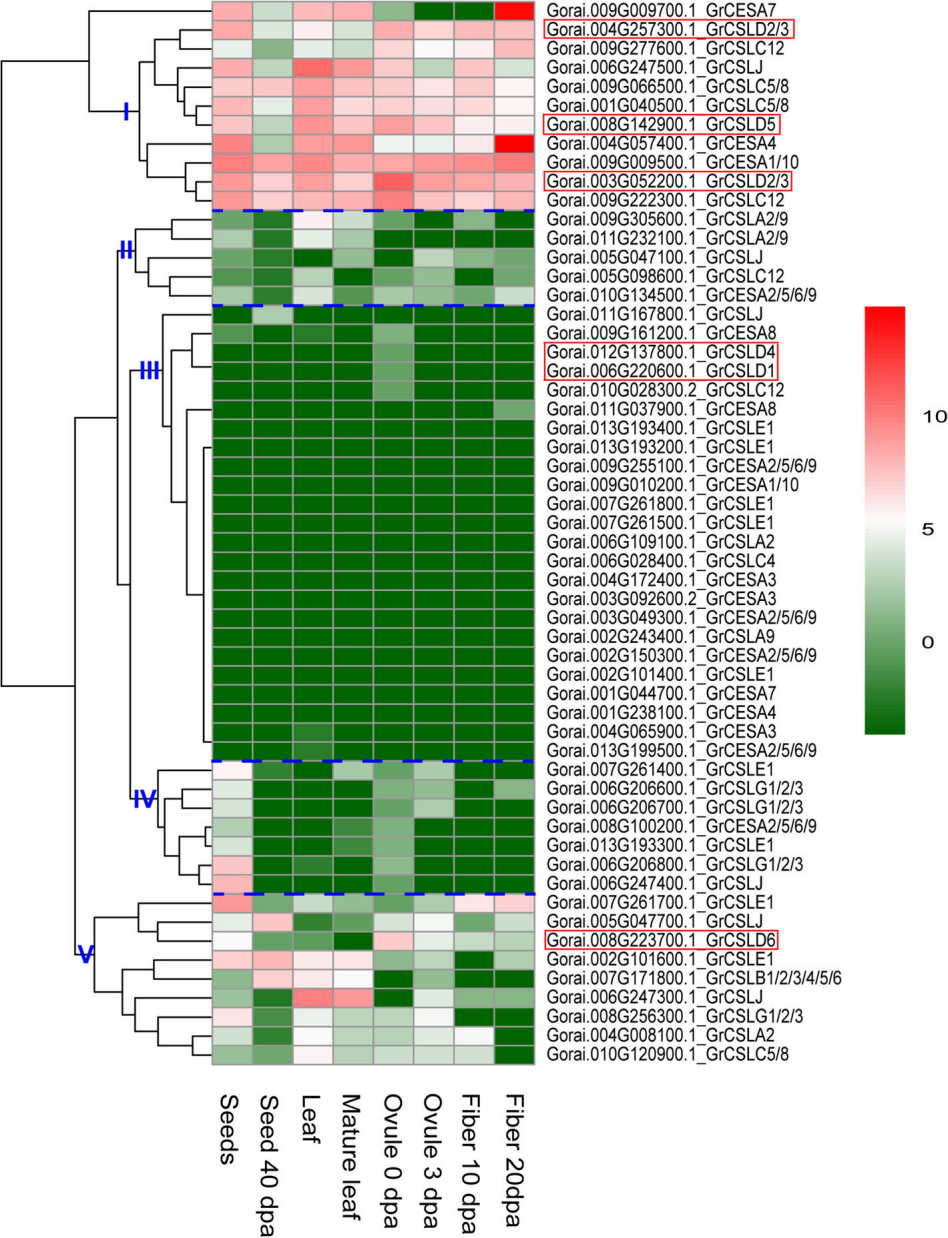
Expression profiling of CESA/CSL gene in *G. raimondii*. The color key representing the count data that were subjected to variance stabilization transformation in the DESeq packages is shown right. Red, white and green refers to high expression, medium expression and low expression, respectively. I, II, III, IV and V denote five major groups based on the hierarchical clustering analysis using pheatmap

Gene expression profiling can provide useful information for understanding gene function. To indicate whether *CSLD* genes have unique function among *CESA/CSL* superfamily, we performed the gene expression and qRT-PCR analysis. The previous report has shown that *OSCESA* genes are highly expressed in most of the tissues examined, and *OsCSL* genes have the rather variable expression [13]. Based on the hierarchical clustering analysis, the *CESA/CSL* gene superfamily can be divided into five major groups in *G. hirsutum*, *G. arboreum* and *G. raimondii* (Figs. 4, 5 and 6). *CSLD1* and *CSLD4* exhibited high expression level in androecium & gynoecium (A&G) in the groups II of *G. hirsutum*. However, transcripts of *CSLD1* and *CSLD4* were zero or very small scores in other tissues of *G. hirsutum*, *G. arboreum* and *G. raimondii* (Figs. 4, 5 and 6, Additional file 6: Table S10, S11 and S12). *CSLA2*, *CSLB*, CSLG, *CSLJ*, C*ESA1/10*, *CESA3* and *CESA4* were expressed in A&G of *G. hirsutum*, but these genes, except *CotAD_11650_GhCSLJ*, also showed expression in other tissues (Fig. 4, Additional file 6: Table S10). This result indicated that expression of *CSLD1* and *CSLD4* appeared to have strong specificity, which was similar to the report that *OSCSLD3* and *5* (the orthologs of *A. thaliana CSLD1* and *CSLD4*) showed strong expression in stamen (pollen) in rice and *A. thaliana* [13]. *CSLD5* was primarily expressed at seedlings, root (radicle), stem, leaf and ovule, and other *CSL* genes were also expressed in these tissues (Figs. 4, 5 and 6, Additional file 6: Table S10, S11 and S12). *GhCSLD6* expression was observed in all tissues, and had the low expression in old leaves, bract, ovule 30 dpa and ovule 40 dpa (Fig. 4, Additional file 6: Table S10). *CSLD 6* was expressed strongly in fiber, consistent with a previous report [21, 45]. However, *GrCSLD6* only exhibited strong expression at ovule (Fig. 6). *GhCSLD2/3* genes fell into three distinct groups (in V), unlike *GhCSLD1*, *4* and *5*, which were divided into one group (Fig. 4). *CSLD2/3* showed primary expression in seed, seedlings, cotyledon, root, stem, leaf, corolla, ovule, fiber and boll shell (Figs. 4, 5 and 6, Additional file 6: Table S10, S11 and S12). *CSLD2/3* genes in three distinct groups (two distinct groups in *G. arboreum* and *G. raimondii*) showed the different pattern of expression (Figs. 4, 5 and 6). These results implied that *CSLD2/3* had multiple functions in synthesis of cell walls at the different development stages.

**Fig. 7:**
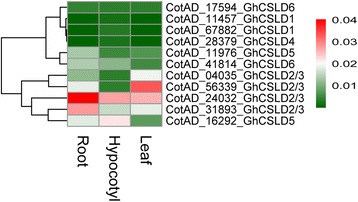
Heat map of qRT-PCR in *G. hirsutum*. The color key representing relative expression level of *CSLD* genes by comparative 2^-ΔΔCT^ method is shown right. Red, white and green refers to high expression, medium expression and low expression, respectively

Generally, *CESA* genes showed an extensively high expression in all the tissues examined (Figs. 4, 5 and 6). *Gh(a)CESA2/5/6/9*, *1/10* and *3* were expressed during primary cell wall biosynthesis at all tissues. *Gh(a)CESA4, 7* and *8* were strongly expressed in secondary cell walls of tissues, for example, fiber 20 dpa. The expression patterns of *CESA* genes were similar to those of the *OSCESA* and *ATCESA* [13]. All of *CSL* genes showed relative tissue-specific expression, unlike *CESA*, which were expressed constitutively. The total expression of *GhCSLA* genes was highest in cotyledon Y1, and was followed by high expression in boll shell, with the lowest expression detected in the cotyledon Y2. In contrast*,* the total expression of *GhCSLB* genes was highest in cotyledon Y2. The total expression of *GhCSLC*, *D, E, G* and *J* genes was highest in stem Y1, A&G (mostly contributed by *CSLD1* and *CSLD4*), root, cotyledon Y1 and cotyledon Y2, respectively (Additional file 6: Table S10). The total expression of *GaCSLA* and *E* genes was highest in seedling. The total expression of *GaCSLB* and *G* genes was highest in seed. The total expression of *GaCSLC* was highest in seed 40 dpa. The total expression of *GaCSLD* (mostly contributed by *CSLD5*) was highest in leaf (Additional file 6: Table S11). *GrCSLA* genes showed an almost undetectable expression in all tissues. The total expression of *GrCSLB* was highest in seed 40 dpa. The total expression of *GrCSLC* and *J* was highest in mature leaf. The total expression of *GrCSLD* (mostly contributed by *CSLD2/3*) was highest in ovule 0 dap. The total expression of *GrCSLE* and *G* was highest in seed (Additional file 6: Table S12). These results indicated that the expression of the *CSL* genes of the whole family often accumulated to high levels in one or more of the tissues for that the *CSL* members showed preferences, which were similar to a previous report in rice [13]. To gain more insights into whether the expression of *CSLD* genes is different, we performed qRT-PCR experiments with specific primers in *G. hirsutum* (Additional file 7: Table S13). Among all the 11 analyzed *GhCSLD* genes, one copy of *GhCSLD2/3* (CotAD_24032) had the most prominent expression levels in all tissues (Fig. 7), followed by *GhCSLD2/3* (CotAD_56339), *GhCSLD2/3* (CotAD_31893) and *GhCSLD5* (CotAD_16292). *GhCSLD1* and *GhCSLD4* showed an almost undetectable expression in all the tissues. *GhCSLD6* was expressed at moderate levels. Overall, the results from the qRT-PCR expression data closely agreed with those of RNA-seq (Fig. 4). The results showed that *CSLD* genes exhibited the different pattern of expression compared with other *CSL* genes, and all the *CSLD* genes were differentially expressed in different cotton tissues under normal growth conditions, which indicated the functional diversification of *CSLD* genes in cotton.

### Identification of positive selection on the GrCSLD1 protein

Positive selection increases the frequency of mutations that confer a new fitness advantage to individuals carrying those mutations [50]. For protein-coding DNA sequences, positive selection is indicated by a ratio of nonsynonymous/synonymous mutation rates (ω = dN/dS) greater than one [51]. Positive selection might occur if the gene is involved in plant-pathogen competition [52], if new and beneficial function emerged at the point of duplication [53], in response to stress [54], etc.

A site can be defined as undergoing long-term positive selection if it experiences positive pressure in most or all branches of the phylogeny [54, 55]. We only focused on those branches defining the major clades of cotton, denoted CSLD1 to CSLD6 (Fig. 3). We used the Notung method [56] to infer gene duplication and determined long-term positive selection after duplication by applying the branch-site model at the clade level (across all branches in each specified clade). Two branches (CSLD1 and CSLD4) exhibited episodic positive selection after Bonferroni correction. Nine and five sites were identified as undergoing positive selection after duplication by the Bayes empirical Bayes analyses, respectively (Table 2). CSLD1 and CSLD4 also showed a long-term shift in positive selection across every branch of the cotton lineage. Thirteen and two sites with significant evidence for positive selection were detected in the CSLD1 and CSLD4 clades, respectively. Amino acid residues of positive sites are shown in Table 2.

**Table 2:** Amino acid sites using the branch-site model under positive selection

Foreground	*ω*	*P*-value	Site under positve selection
CSLD1 branch	7.91	*P* < 0.001	S29*, K116*, C157*, A244*, C795*, Q894*, A912*, C917*, C947*
CSLD1 clade	3.56	*P* < 0.001	D2**, N6*, S8*, S29*, K116*, C157*, N188*, A244*, C795*, Q894*, A912*, C917*, W952*
CSLD4 branch	5.39	*P* < 0.001	T314*, F929*, Q953*, G1046*, G1104*
CSLD4 clade	2.57	*P* < 0.001	Q953*, G1104*

Sites are numbered according to the full *GrCSLD1* coding sequence. Sites with posterior probabilities greater than 0.95 (*) and 0.99 (**) are shown

The positively selected sites in Table 2 were located on the predicted tertiary structure of the GrCSLD1 protein. K116 is spatially close to the class-specific region (CSR), and A244 is located on the β1-strand (Additional file 8: Figure S3 and Additional file 9: Figure S4). C157, C795, Q894, A912, C917 and C947 are positioned in the transmembrane helices (TMHs). Furthermore, C795, A912 and C917 are located within transmembrane pore predicted by MEMSAT (Additional file 8: Figure S3 and Additional file 9: Figure S4).

### Structure of GrCSLD1 protein

In *A. thaliana*, *CSLD1* and *CSLD4* are both expressed at high levels in the pollen tubes and mature pollen grains, and the synthesis of the pollen tube wall is significantly reduced in *CSLD1* and *CSLD4* mutants [30]. The CSLD1 and CSLD4 proteins are localized in the Golgi apparatus before germination and are then transported to the plasma membrane at the pollen tube tip [30, 34]. These results suggest that the CSLD1 and CSLD4 proteins probably present distinct cellulose synthesis activities at the apical plasma membrane during tip growth in pollen tube cells. To gain insights into the function of CSLD1 proteins in cotton, a structural model was built via template-based and template-free modeling.

I-TASSER, Phyre2 and Robetta (prediction of domains with comparative modeling, see Methods) were all used with BcsA [57] as the primary template. The model validation scores of the full-length GrCSLD1 protein are shown in Additional file 10: Table S15. We identified the top-scoring model predicted by Robetta. Structural alignment of the top-scoring model with BcsA gave a TM-score of 0.65, suggesting that GrCSLD1 and BcsA share the same fold [58].

All known CSLD proteins are classified as GT2 family in the CAZy database [9]. GT2 proteins are predicted to be inverting enzymes, that is, the configuration of the anomeric sugar carbon is inverted during the transfer reaction [59]. The GT2 family includes cellulose synthase, β-1,4-mannan synthase, and chitin synthase. The GT domain has a GT-A fold consisting of seven α-helices, three amphipathic interface (IF) helices (IF1-3) attached to the transmembrane region, and a seven-stranded β-sheet that resembles a Rossmann fold [57, 60]. However, a three-dimensional structure of a cotton CSLD protein has not been solved.

**Fig. 8:**
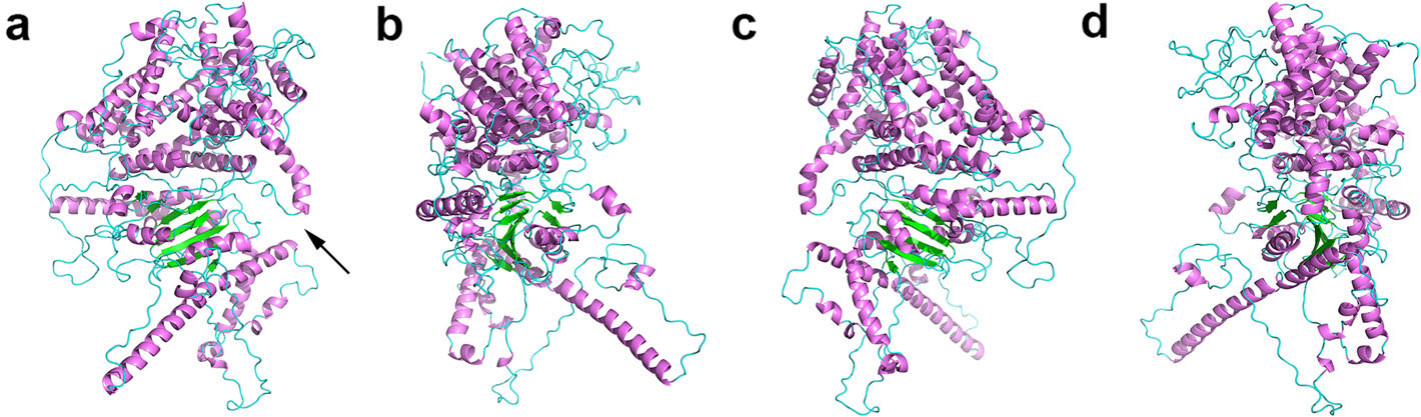
Structural model of GrCSLD1. The indicated structures are progressively rotated 90° from left to right (**a**-**d**). The *black arrow* indicates the active site motifs in (**a**)

**Fig. 9:**
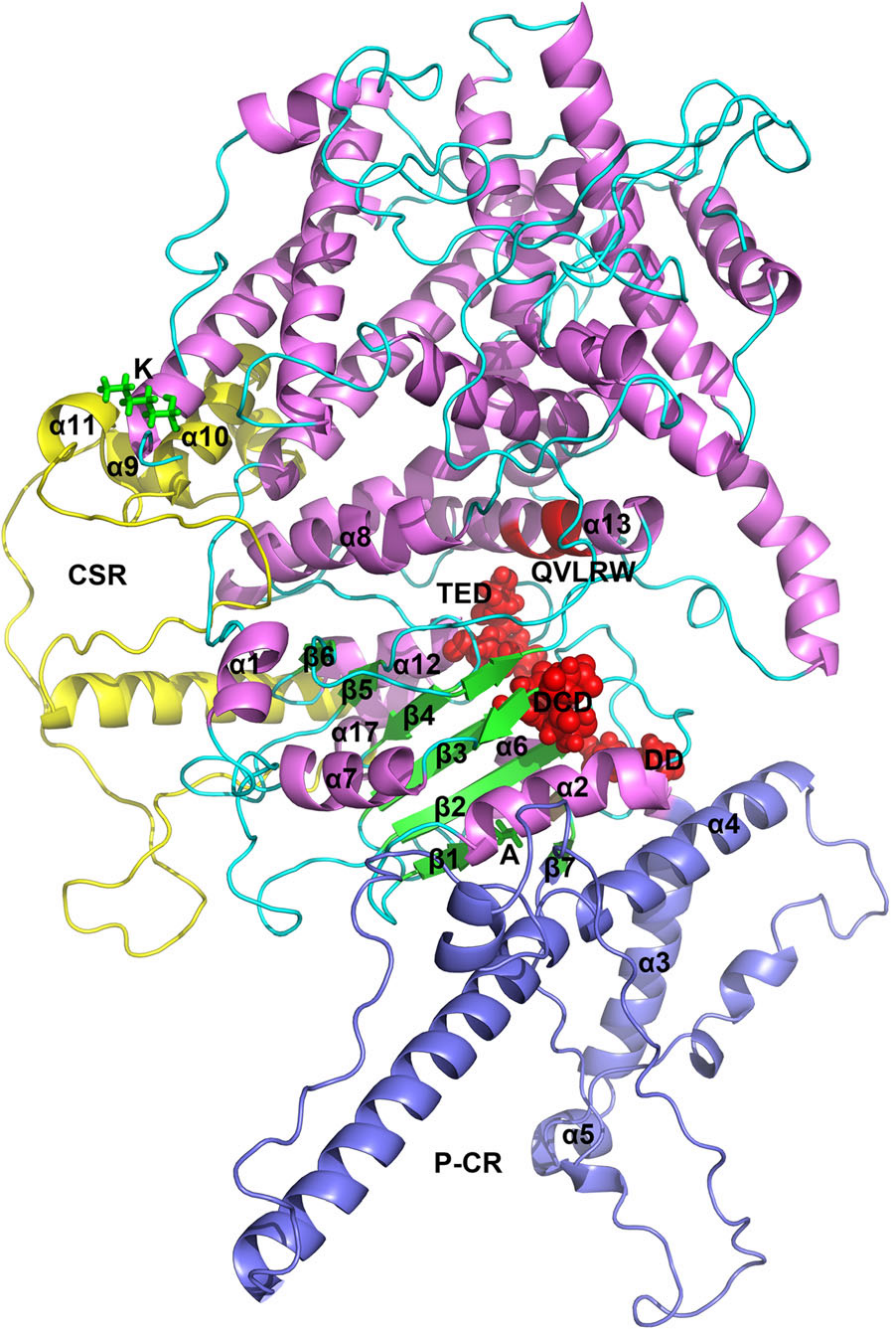
Structural model of GrCSLD1 showing the positions of the amino acid residues under positive selection, plant-specific regions and active site motif. The structures of GrCSLD1, P-CR and CSR are colored violet, light blue and yellow, respectively. The core domain contains 8 α-helices (α2, α4, α6, α7, α8, α12, α13, and α17) and the seven-stranded β-sheet. The numbering of the α-helices and β-strands is based on their order in the secondary structure of GhCESA1 (Additional file 9: Fig. S4). Red highlights DD, DCD, TED (spheres) and QVLRW. The sites (K and A) under positive selection are shown as green sticks

**Fig. 10:**
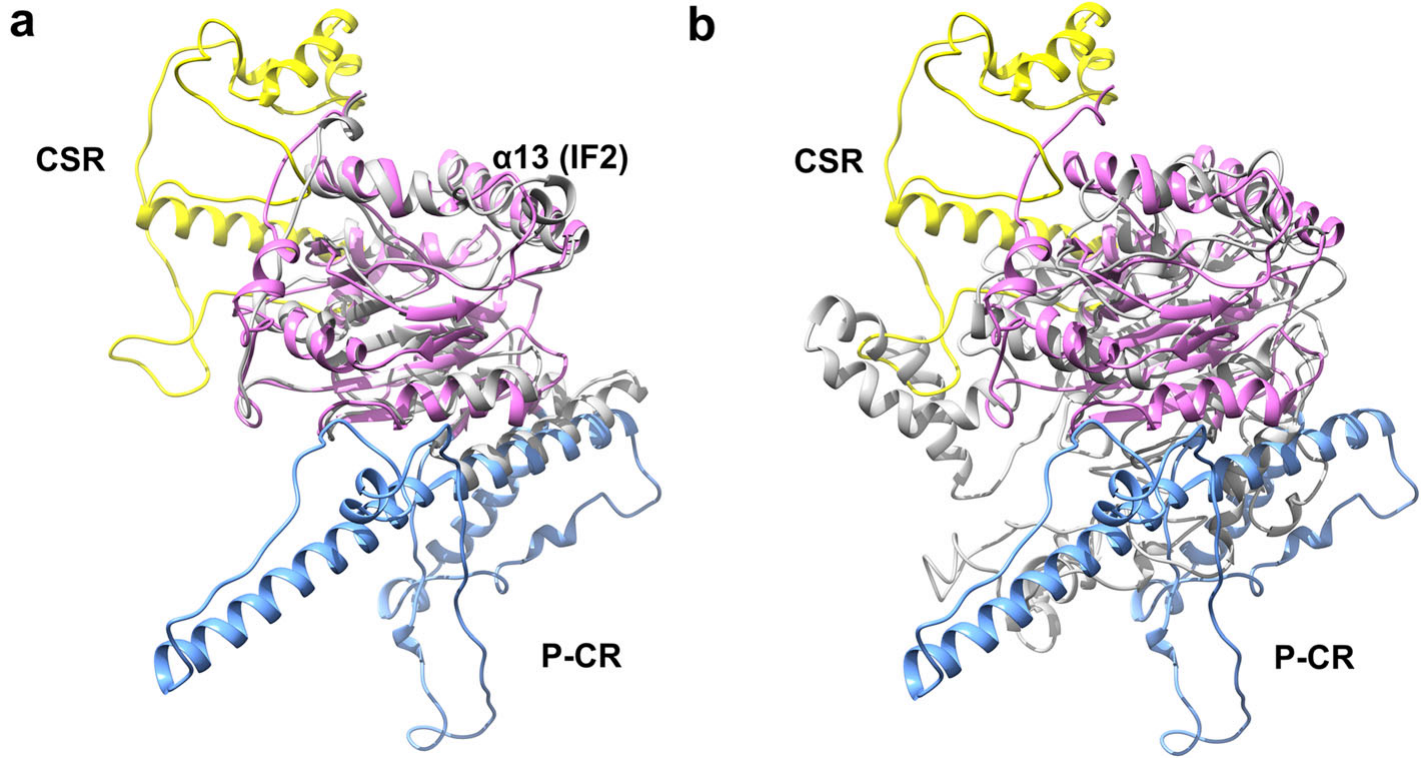
Superimposition of the core domain of GrCSLD1 with BcsA and GhCESA1. Residues 171 to 727 of GrCSLD1 were superimposed with residues 119 to 394 of BcsA (**a**) and residues 220 to 725 of GhCESA1 (**b**) using MatchMaker in UCSF-Chimera. All proteins adopt a GT-A fold. **a** The overall RSMD value between matched C_α_ atoms was 1.84 Å, and α13 denotes the motif corresponding to IF2 of BcsA. **b** The overall RSMD value between matched C_α_ atoms was 2.74 Å. GrCSLD1, BcsA and GhCESA are colored violet, gray and gray, respectively. P-CR and CSR are indicated in light blue and yellow

Our results show that the predicted GrCSLD1 structure contains 31 α-helices and 9 β-strands (Figs. 8 and 9, Additional file 9: Figure S4). The GrCSLD1 core domain was superimposed with GhCESA1 [61] and BcsA [57] using MatchMaker in UCSF-Chimera [62]. In the superimposition of GrCSLD1 with BcsA and GhCESA1, structure matching of the domain included 8 helices (α2, α4, α6, α7, α8, α12, α13, α17) and 7 β-strands that form the β-sheet (β1, β2, β3, β4, β5, β6, β7), with an overall root mean square deviation (RMSD) of 1.84 Å, and 6 helices (α2, α6, α7, α8, α12, α13) and 6 β-strands that form the β-sheet (β1, β2, β3, β4, β5, β6) with an overall RMSD of 2.74 Å, respectively (Figs. 9 and 10, Additional file 9: Fig. S4). Therefore, the core domain of GrCSLD1 contains 8 α-helices (α2, α4, α6, α7, α8, α12, α13, α17) and the seven-stranded β-sheet that forms a Rossmann fold (Fig. 9, Additional file 9: Figure S4). The core domains of GrCSLD1 and GhCESA1 show structural congruence, even though the GhCESA1 structure was not used for prediction of the GrCSLD1 model. By analogy to GhCESA1, the catalytic pocket of GrCSLD1 comprises the closely arranged, conserved DD, DCD, TED and QVLRW motifs. The α13 helix is positioned near these conserved motifs, corresponding to IF2, which interacts with the cellulose acceptor substrate (Fig. 10a) in BcsA [57]. GrCSLD1 only contains a cellulose_synt domain (Fig. 1), and the *CSLD2/3* genes also have been suggested to be involved in mannan synthesis during cotton fiber cell development [47]. These results suggest that GrCSLD1 belongs to GT2 and probably participates in the biosynthesis of cellulose, mannan or other polysaccharides.

## Discussion

The CSLD proteins, which feature a conserved D, D, D, QXXRW motif, belong to the ancient cellulose synthase superfamily [10, 12]. In addition to CESA, the CSLD proteins are the only members of the superfamily with a zf-RING (Fig. 1) domain in the N-terminal region, which is thought to function in protein-protein interactions [12]. The CSLD proteins remain poorly understood despite their importance for tip-growing cells and stem growth.

### Conserved synteny of *CSLD* genes distributed across the cotton genome

We identified 23 full-length CSLD proteins: six, six and 11 from *G. arboreum, G. hirsutum* and *G. raimondii*, respectively (Table 1). The *CSLD* genes are distributed across several chromosomes. Conserved synteny of all *CSLD* genes was observed between *G. arboreum* and *G. hirsutum*. These are one-to-two syntenic relationships, except for *CSLD4* genes on Chr4, which had a one-to-one syntenic relationship between *G. arboreum and G. hirsutum* (Fig. 2). The one-to-one syntenic relationship of *CSLD4* genes exists because *CSLD4* is a single-copy gene in *G. hirsutum*. However, conserved synteny of *CSLD1* genes was not detected between *G. raimondii* and *G. hirsutum* (Fig. 2). There are one-to-two syntenic relationships for *CSLD4*, *CSLD5*, *CSLD6* and one copy of *CSLD2/3*. No syntenic relationships were identified for *CSLD1*, perhaps because the synteny hits are concealed by the annotation string search in SyMAP [63]. The one-to-three syntenic relationships in one copy of *CSLD2/3* on Chr03 between *G. raimondii* and *G. hirsutum* might be caused by two closely related isoforms (ATCSLD2/3) in *A. thaliana*. One-to-two syntenic relationships were mostly identified between *G. arboreum* or *G. raimondii* and *G. hirsutum* because the *G. hirsutum* genome is derived from hybridization of A_2_ and D_5_ genome ancestors [45]. Comparison of the synteny map and CSLD phylogeny showed that most *CSLD* genes in synteny blocks form a monophyletic clade, indicating that *CSLD* genes have been conserved over considerable time, whereas genes within the clades have evolved.

Recent studies have shown that the present allotetraploid *G. hirsutum* was derived from hybridization of A_2_ and D_5_ genome ancestors approximately 1.5 MYA [45]. The *G. arboreum* and *G. raimondii* genomes have undergone two rounds of whole-genome duplication (WGD), which are estimated to have occurred approximately 13-20 and 115-146 MYA, respectively [43, 48]. The ancient duplication event corresponds to the ancient hexaploidization event shared among eudicots [64]. *G. arboreum* and *G. raimondii* have approximately the same number of CSLDs as *A. thaliana*, *Oryza sativa* and *Zea mays*, and *G. hirsutum* has approximately twice as many as *G. arboreum* or *G. raimondii*. Moreover, the CSLD proteins of cotton and those of *Physcomitrella patens* and *Selaginella moellendorffii* form a sister group to CSLD5. These results also suggest that *CSLD* genes are conserved in cotton and an ancient gene family, and the expansion of *CSLD* genes is associated with WGD.

### Reconstructing phylogenetic trees of CSLD proteins

The quality of the multiple sequence alignment (MSA) has a profound impact on the robustness of a given phylogenetic tree [65]. Because genes evolve at different rates, some regions of an alignment are very well conserved and suitable for phylogenetic analysis, whereas others are full of gaps and very divergent. These divergent regions cannot be precisely aligned and thus must be removed prior to phylogenetic analysis [66]. Phylogenetic reconstruction produces an estimate of the true history by examining alternative trees and then quantifying the extent to which sequence data support or reject different phylogenetic results. Maximum likelihood [67] and Bayesian inference [68] are the most popular methods to build phylogenetic trees. Therefore, we used multiple alignment strategies (Kalign, Mafft and Muscle), support measures (SH-like approximate likelihood ratio tests, non-parametric bootstrap proportions and Bayesian posterior probabilities) and alignment trimming (Gblocks) in the current study.

Our results suggest that the cotton CSLD phylogenetic trees inferred from ML and Bayesian, based on three alignments and an elision strategy can be divided into five strongly supported clades. The division of the phylogenetic tree of the CSLD proteins into five clades is also robust with respect to other factors that are known to affect phylogenetic tree accuracy, including statistical-support measures and evolutionary models. However, the topology of the cotton CSLD5 and CSLD2/3 trees inferred from ML and Bayesian, based on two alignments (Kalign, Mafft) and the elision strategy, had some differences (Additional file 5: Figure S1). The Bayesian trees of CSLD5 and CSLD2/3 trees based on two alignments (Kalign and Mafft) have polytomies. We consider these soft polytomies because the trees from other methods were fully binary. The appearance of polytomies may be due to contradictory results from conflicting data and a lack of information regarding the true bifurcating pattern of the proteins [69]. The ML and Bayesian trees based on Muscle and elision alignments showed almost identical topology and best estimate the true evolution of CSLD proteins. We used the ML tree based on Muscle (bootstrap branch supports) to infer duplication and evaluate positive selection. Cotton *CSL* genes are involved in the synthesis of cell wall matrix polysaccharides surrounding cellulose microfibrils in cotton [20]. *CSLD2/3* and *CSLD6* but not *CSLD1* and *CSLD4* genes are expressed strongly during fiber development [21, 45]. The CSLD2/3 proteins also have been suggested to be involved in mannan synthesis during cotton fiber cell development [47]. These results imply that CSLD proteins may participate in the biosynthesis of cellulose, mannan or other polysaccharides.

### Characterization of *CSLD* gene family

*A. thaliana* and rice provide a reference point for understanding the function of cotton CSLD proteins. CSLD proteins in *A. thaliana* might be involved in cellulose synthesis in tip-growing cells (pollen tubes and root hairs), stem growth and mannan synthesis, which suggests that CSLD proteins have acquired different functions.

To demonstrate the functional characterization of *CSLD* genes, we performed the gene expression and qRT-PCR analysis. The previous report has shown that *OSCESA* genes are highly expressed, and *OsCSL* genes have the rather variable expression [13]. Almost all *CESA* genes in cotton exhibited high expression in all tissues examined, implying that their major roles in the biosynthesis of cellulose, the core structural component of the cell wall. *CESAs* (*2/5/6/9*, *1/10* and *3*) and *CESAs* (*4, 7* and *8*) were strongly co-expressed (in IV and V group), suggesting that *CESAs* (*2/5/6/9*, *1/10* and *3*) and *CESA* (*4, 7* and *8*) may form two synthesis complexes involved in primary and secondary cell wall synthesis, as observed in the model plant *A. thaliana* [2, 12] and rice [13]. The results also were consistent with the report that CESA1, 2, 7, 8 (the orthologs of *A. thaliana* CESA8, 4, 7 and 7, respectively) are associated in the cellulose biosynthesis secondary cell wall, whereas CESA3, 5, 6, 9 and 10 (the orthologs of *A. thaliana* CESA3, 2/5/6/9, 1/10, 2/5/6/9 and 3, respectively) participate in primary cell wall synthesis in cotton [19–22].

One copy of *GrCESA1/10* gene exhibited high expression in all tissues, and one copy of *GrCESA4* and *7* was strongly expressed in fiber 20dpa, which suggesting that there are fewer GrCESA proteins involved in the biosynthesis of cell wall (Fig. 6, Additional file 6: Table S12). Compared with *CESA* genes, *CSLD* genes were expressed in one or more of the tissues.

In *CSL* gene superfamily, The total expression of *CSLD* genes was different from other *CSL* genes. Furthermore, different copy of *CSL* genes showed different expression patterns. *CSLD1* and *CSLD4* were strongly co-expressed in A&G, and showed tissue-specific expression (Figs. 4 and 7), suggesting that *CSLD1* and *CSLD4* may form a synthesis complex involved in polysaccharides. The overall expression of *CSLD2/3* and *CSLD5* genes was highest in root and leaf, respectively (Additional file 6: Table S10). These results were consistent with the previous reports in *A. thaliana* and rice [13, 30, 34]. *CSLD 6* was expressed strongly in fiber, consistent with a previous report [21, 45]. *GrCSLD6* only exhibited expression at ovule (Fig. 6); however, *ATCSLD6* appears to be a pseudogene [31]. These results imply the *CSLD* genes show relative tissue-specific expression, indicating their potentially different function in the biosynthesis of polysaccharides.

### Spatial distribution of amino acids under positive selection in GrCSLD1

Branch-site model analyses showed differences in the selection pressure on major clades, which implies that some sites in CSLD proteins from cotton are subject to different constraints during the evolutionary process. These constraints are imposed by the varied functional roles and evolutionary origins of CSLD proteins. CSLD2/3, CSLD5 and CSLD6 were found to have undergone relaxed purifying selection. However, *CSLD1* and *CSLD4* showed episodic positive selection and long-term shift positive selection across every branch of the cotton lineage after gene duplication. *CSLD1* (*ATCSLD4*), *CSLD2* and *CSLD4* (*ATCSLD5*) showed a strong positive selection signal in grasses [54]. It is possible that there are different evolutionary pressures in cotton and grass. The *CSLD1* and *CSLD4* genes are required for normal growth of pollen tubes in *A. thaliana*, possibly by participating in pollen tube cellulose synthesis [30, 34]. The gene expression and qRT-PCR analysis showed that *CSLD1* and *CSLD4* genes only exhibited strongly expression in A&G, and have the different expression patterns from other *CSLD* genes, which imply that CSLD1 and CSLD4 have the potentially different function in the biosynthesis of polysaccharides, compared with other CSLD proteins. Our results suggest that *CSLD1* and *CSLD4* genes probably evolved new functions after gene duplication through long-term shifts in positive selection.

The recently reported three-dimensional structure of the A and B subunits of a bacterial cellulose synthase complex from *Rhodobacter sphaeroides* [57] and a computational model of cotton GhCESA1 [61] provide an opportunity to define the three-dimensional distribution of the positively selected sites in GrCSLD1. However, the distinct functions of CSLD proteins remain unknown. Some reports have shown that CSLD proteins are associated with cellulose and mannan biosynthesis [29–31, 47]. There is no direct evidence that the GrCSLD1 protein has a distinct catalytic function. Functional characterization based on the predicted three-dimensional structure of GrCSLD1 proteins is extremely difficult. GrCSLD1 contains a conserved cellulose_synt domain (Fig. 1) and exhibits a phylogenetic relationship with other functionally known CSLD proteins in other plants and structural similarity with BcsA and GhCESA1. The three-dimensional structure of GrCSLD1 is predicted to contain a Rossmann fold and has a conserved D, D, D, QXXRW motif (Fig. 9). These results imply that GrCSLD1 belongs to GT2; however, the definite role of GrCSLD1 is not known. We only suggest GrCSLD1 probably participates in the biosynthesis of cellulose, mannan or other polysaccharides.

The amino acid residues identified as under positive selection in the CSLD1 lineage are located on a region adjacent to the CSR, β1-strand and TMHs in the structure of GrCSLD1 (Fig. 9, Additional file 8: Figure S3 and Additional file 9: Figure S4). The structure of GrCSLD1 revealed that CSR and P-CR fold into distinct subdomains within the cytosolic region. The CSR region probably helps stabilize CESA assembly through non-covalent interactions [61]. K116, a residue under positive selection that is spatially adjacent to the CSR region, may help stabilize CSLD1 assembly into complexes with other CSLDs (*CSLD1* and *CSLD4* genes are strongly expressed in A&G), similar to CESAs. Interestingly, A244 is positioned on a β1-strand within the core domain of GrCSLD1, which suggests that this residue has the potential to influence GrCSLD1 activity. C157, C795, Q894, A912, C917 and C947 are positioned in the predicted TMHs and within the transmembrane pore that is involved in the extrusion of the nascent polysaccharide across the cell membrane (Additional file 8: Figure S3 and Additional file 9: Figure S4). Our analyses suggest that the residues of GrCSLD1 under positive pressure have relatively significant influence on enzyme activity or function, and on the fine structure of the polysaccharide that enzyme synthesizes. The specific roles of these sites under positive selection in GrCSLD1 remain unknown and warrant further research.

Recent reports show that CSLD proteins are not included in the modules of cell wall polymer biosynthesis in rice [70] and that the CSLD proteins are not interacted with cellulose synthase complexes in cotton [71]. *CSLD1* and *CSLD4* genes may be specifically involved in biosynthesis of cellulose at the tip of growing pollen tube and are highly expressed in mature pollen grains and pollen tubes in *A. thaliana* [30]. These results also suggest that CSLD1 and CSLD4 probably function as a complex in cellulose biosynthesis.

## Conclusions

The CSLD family remains relatively uncharacterized within the community, and many questions about its evolutionary history and function remain. In this study, we performed rigorous phylogenetic analyses with maximum likelihood and Bayesian methods to resolve the phylogenetic topology of CSLD proteins in cotton. Tests for positive selection, gene expression profiling and qRT-PCR analysis were performed in the context of determining characterization of *CSLD* genes, compared with *CESA* and other *CSL* genes. These analyses were supplemented with GrCSLD1 homology modeling to provide a structural context for the evolutionary and functional characterization of CSLD proteins. These data provide a basis for understanding the evolutionary history and 3D modeling of CSLD proteins in cotton.

## Methods

### Identification of CSLD proteins

We used confirmed functional protein sequences of CSLD in *A. thaliana* as queries to identify new CSLD protein homologs from fully sequenced genomes of cotton (*G. arboreum, G. hirsutum and G. raimondii*) and 15 other plant species (Additional file 1: Table S1) using BLASTP (E-value ≤1E-5) [72]. The *G. arboreum* and *G. hirsutum* sequences were retrieved from CGP (http://cgp.genomics.org.cn). *G. raimondii* and 15 other fully sequenced plant genomes were retrieved from Phytozome (V11) [73]. To distinguish CSLD from CESA and other CSLs, we used the hit candidates of CSLD to search against the proteome of *A. thaliana* from Phytozome using BLASTP. Each true CSLD is expected to identify a CSLD from *A. thaliana* as the top hit according to the nomenclature of the cellulose synthase superfamily of *A. thaliana* [12]. *CESA* has more exons than *CSLD*. The conserved domains of all obtained sequences were verified via sequence searches with the online program SMART [74], Interpro [75] and the NCBI conserved domain databases [76]. Synteny blocks between *G. hirsutum* and *G. arboreum* or *G. raimondii* were detected using SyMAP by default [63] and visualized with Strudel [77].

### Multiple sequence alignment

The CSLD protein sequences were aligned using Kalign v2.04 with default parameters [78], E-INS-I methods from Mafft v7.215 [79], and Muscle v3.8.31 [80]. Divergent and ambiguously aligned regions from the resulting alignments were trimmed with Gblocks v0.91b [81] prior to phylogenetic analysis. We also produced an elision alignment by concatenating all three individual Gblocks-processed alignments [82].

### Phylogenetic analysis

Maximum likelihood phylogenetic trees were reconstructed using PhyML v3.0 [83], with the best-fit models of amino acid substitution selected by ProtTest v3.2 [84]. Branch supports were estimated using SH-like approximate likelihood ratio tests [85] and non-parametric bootstrap proportions (500 replicates). Bayesian phylogenies were reconstructed using MrBayes v3.2.5 [49]. We integrated out amino acid substitution models (prset aamodelpr = mixed) and assumed a model of discrete-gamma distributed rate variation across sites. The Markov chain was sampled every 100th generation, and the initial 25% of samples were discarded as burn-in, with the remaining samples used to generate the consensus tree. We assessed chain convergence by running two simultaneous, independent analyses and terminated the analysis when the average standard deviation of split frequencies between the two runs fell below 0.01. Phylogenetic trees obtained from ML and Bayesian reconstructions were compared regarding both topology and branch lengths using Ktreedist [86].

### Gene expression and qRT-PCR analysis

The high-throughput RNA-sequencing data were downloaded from Short Read Archive of the National Center for Biotechnology Information (http://www.ncbi.nlm.nih.gov/sra, Additional file 11: Table S4, S5 and S6). The RNA-seq reads were mapped to the reference cotton genome with TopHat2 [87]. The set of files containing mapped reads from TopHat2 were sorted and indexed using samtools [88]. The overlap of reads with genes were counted using HTseq-count [89]. The counts of genes were estimated normalization and dispersions, and were transformed to variance stabilization data with DESeq (Additional file 12: Table S7, S8, and S9) [90, 91]. We produced the heatmaps based on the variance stabilization transformed data for *CESA/CSL* gene superfamily of cotton using pheatmap package (pheatmap: Pretty Heatmaps, R package version 1.0.8, https://CRAN.R-project.org/package=pheatmap). We used PF03552 (Cellulose_synt) and PF00535 (Glycos_transf_2) as queries to identify new CESA/CSL protein homologs from fully sequenced genomes of cotton (*G. arboreum*, *G. hirsutum* and *G. raimondii*) using HMMER 3.1b2 package [92]. The CESA/CSL protein sequences were aligned using Muscle v3.8.31 [80]. Divergent and ambiguously aligned regions from the resulting alignments were trimmed with Gblocks v0.91b [81] prior to phylogenetic analysis. Maximum likelihood phylogenetic tree was reconstructed using PhyML v3.0 [83], with the best-fit model (JTT + G + F) selected by ProtTest v3.2 [84]. The CESA/CSL protein sequences of *A. thaliana* were downloaded from the TAIR 10 database (https://www.arabidopsis.org) [93]. All the identified *CESA/CSL* genes in cotton (*G. arboreum*, *G. hirsutum* and *G. raimondii*) were provided specific names based on the orthologous sequence with *A. thaliana* (Additional file 13: Figure S2). *G. hirsutum* (Chinese cotton cultivar Yinshan 2, Henan Qiule Seed Industry Science&Technology LTD., COM) were grown in a growth chamber at 28 °C with a 14 h light and 10 h dark cycle. When three fully expanded leaves appeared, root, hypocotyl and leaf were collected separately, frozen immediately in liquid nitrogen and stored at −80 °C until RNA extraction. Each sample was preformed in three biological replicates. Total RNA was extracted from root, hypocotyl and leaf using Trizol reagent according to the manufacturer’s instructions (TaKaRa), and treated extensively with RNase-free DNase I. The cDNA was synthesized from 1 μg of total RNA using a First Strand cDNA Synthesis Kit (Invitrogen). The primers of *CSLD* genes from *G. hirsutum* designed for the qRT-PCR analysis are listed in Additional file 7: Table S13. QRT-PCR was performed as previously described ﻿ [94, 95]. The comparative 2^-ΔΔCT^ method was used to calculate the relative expression level of *CSLD* genes (Additional file 14: Table S14) [96]. The heatmap for the qRT-PCR analysis was generated by pheatmap package (pheatmap: Pretty Heatmaps, R package version 1.0.8, https://CRAN.R-project.org/package=pheatmap).

### Positive selection

The detection of positive selection in cotton CSLD protein-coding genes across the phylogeny with the branch-site model was implemented in slimcodeml [97, 98]. In this model, the branch in which we test positive selection is called the foreground branch, and all other branches on the tree are called the background branches. We assume that the ω ratio varies among codon sites, and the codon sequence is divided into four site classes. Site class 0 (with proportion *p*_*0*_) includes codons that are highly conserved or evolve under purifying selection on all branches, with 0 < *ω*_*0*_ < 1. Site class 1 (with proportion *p*_*1*_) includes codons that are neutral, with *ω*_*1*_ = 1. Codons in site classes 2a and 2b (with proportion *1- p*_*0*_ *-p*_*1*_) evolve under positive selection, with *ω*_*2*_ > 1, but the background branches are conserved or neutral [99]. We calculated the likelihood of positive selection at each site along the cotton branches using branch-site model A (model = 2, NSsites = 2) versus the corresponding null model. To guard against codeml getting stuck in local maxima, the analysis was conducted in triplicate with varying initial dN:dS [54, 100]. *P* values were estimated using a chi-square distribution with one degree of freedom. Bayes empirical Bayes (BEB) was available for calculating the posterior probability for each site [99]. Sites with BEB posterior probabilities >0.95 were considered under positive selection. To test whether post-duplication selection represented a long-term shift in selective pressure or the evolution of functional differentiation, we performed branch-site model (model = 2, NSsites = 2) analyses at the cotton clade level, considering all branches following the duplication event as the foreground and the remaining branches as background. We additionally corrected for multiple tests using the Bonferroni correction.

### Structural modeling

The secondary structure and TMHs of GrCSLD1 were predicted using the DSS algorithm of PyMOL (The PyMOL Molecular Graphics System, Version 1.7, Schrodinger, LLC) and MEMSAT [100, 101], respectively.

Computational methods for predicting three-dimensional protein structures can generally be divided into two categories, template-based (comparative and threading modeling) and template-free modeling (ab initio modeling), with some composite protocols combining aspects of both [102, 103]. To obtain a refined three-dimensional structure of GrCSLD1, prediction was preformed using I-TASSER [104, 105], Phyre2 [106] and Robetta [107, 108]. Because Robetta uses the Ginzu method to parse the input protein sequences into domains, builds models for domains with sequence homology to proteins of PDB using comparative modeling, and models for domains without a detectable PDB homolog using the Rosetta ab initio protocol, the structure of GrCSLD1 was broken up into two putative domains, which were modeled separately. The domain models of Rosetta were evaluated using the DOPE functions of MODELER [109], Verfity3D [110], ProSA [111]. The top-scoring models of two domains were recombined together using the hybridizeMove function of RosettaCM. Candidate models of the full-length GrCSLD1 were again assessed using the DOPE functions of MODELER, Verfity3D, and ProSA. We used the TM-align structural alignment program to match the top-scoring model to the structure of BcsA [57]. The TM-score has a value in (0,1], and a score higher than 0.5 indicates that two structures share the same fold in SCOP/CATH [58].

## Additional files

Additional file 1: Table S1. Chromosomal locus ID and length of CSLD proteins in other plants. (XLSX 16 kb)

Additional file 2: The sequences of CSLD proteins in all plants. (FASTA 134 kb)

Additional file 3: Table S2. Model selection using ProtTest. (DOCX 23 kb)

Additional file 4: Table S3. Comparison of ML and Bayesian trees based on three alignments (Kalign, Mafft and Muscle) using Ktreedist. (DOCX 33 kb)

Additional file 5: Figure S1. The different topologies of cotton CSLD trees reconstructed from ML and Bayesian based on three alignments and the elision strategy. Support values are shown for *A. thaliana*-cotton and cotton CSLD nodes using different color circles as bootstrap proportions/SH-like aLRT scores/Bayesian posterior probabilities. The cotton CSLD protein clades are indicated by different colors. “Other CSLD” indicates the CSLD proteins from other plant species. (TIFF 2007 kb)

Additional file 6: Table S10, 11 and 12. The *CESA/CSL* gene count data from HTseq-count in cotton. (XLSX 31 kb)

Additional file 7: Table S13. Primers of the *GhCSLD* genes used for qRT-PCR analysis. (XLSX 9 kb)

Additional file 8: Figure S3. Diagram of transmembrane helices (TMHs) and the cytosolic loop in GrCSLD1. The labels within the cytosolic loop and TMHs (1-8) show the approximate locations of the four conserved motifs (black), P-CR (purple), CSR (blue), and amino acid residues under positive selection (red). (TIFF 6688 kb)

Additional file 9: Figure S4. Multiple sequence alignments of GrCSLD1, GhCESA1, BcsA and ATCSLD1. The secondary structure of GrCSLD1 was calculated using the DSS algorithm of PyMOL. The violet cylinders, yellow arrows, and black lines indicate the α-helices, β-strand and coil of GrCSLD1; the red rectangles and yellow rectangles indicate the α-helices and β-strand of GhCESA1, and the red lines and yellow lines indicate the α-helices and β-strand of BcsA. The plant-conserved region (P-CR) and class-specific region (CSR) are highlighted with blue and green lines. Large red letters indicate sites of episodic positive selection in GrCSLD1. (TIFF 4834 kb)

Additional file 10: Table S15. Model validation scores of the full-length GrCSLD1 protein. (DOCX 22 kb)

Additional file 11: Table S4, 5 and 6. The source of transcriptome data from *G. hirsutum*, *G. arboreum* and *G. raimondii*. (XLSX 12 kb)

Additional file 12: Table S7, 8 and 9. The express profiles of *CESA/CSL* gene superfamily with normalization and variance stabilizing transformation using DESeq in cotton. (XLSX 49 kb)

Additional file 13: Figrue S2. Phylogenetic analysis of the CESA/CSL proteins in cotton and *A. thaliana*. The phylogenetic tree was inferred using maximum likelihood. Support values are shown for key nodes as bootstrap proportions. (TIFF 2921 kb)

Additional file 14: Table S14. The relative expression level of *CSLD* genes of *G. hirsutum* by comparative 2^-ΔΔCT^ method using qRT-PCR. (XLSX 10 kb)

## Abbreviations

A&G: Androecium & gynoecium
BEB: Bayes empirical Bayes
CESA: Cellulose synthase
CSL: Cellulose synthase-like
CSLD: Cellulose synthase-like D
CSR: Class-specific region
GT2: Glycosyltransferases family 2
IF: Interface
*irx*: *irregular xylem*
ML: Maximum likelihood
MSA: Multiple sequence alignment
MYA: Million years ago
P-CR: Plant-conserved region
RMSD: Root mean square deviation
TMH: Transmembrane helix
WGD: Whole-genome duplication
