# Supplementary material for: Evolution, gene expression profiling and 3D modeling of CSLD proteins in cotton
Source: BMC Plant Biol. 2017 Jul 10;17:119. doi: 10.1186/s12870-017-1063-x (PMC5504666; doi:10.1186/s12870-017-1063-x)
Supplement: Supplementary file 3 — Model selection using ProtTest. (DOCX 23 kb) [file 12870_2017_1063_MOESM3_ESM.docx]

**Additional file 3: Supplementary Table S2. Model selection using ProtTest.**

| Proteins | Alignment | Model | AIC | AICc | BIC |
| --- | --- | --- | --- | --- | --- |
| CSLD | Kalign | LG+I+G+F | 1.00 | 0.01 | 0.00 |
|  |  | LG+I+G | 0.00 | 0.99 | 1.00 |
|  | Mafft | LG+I+G+F | 1.00 | 1.00 | 0.00 |
|  |  | LG+I+G | 0.00 | 0.00 | 1.00 |
|  | Muscle | LG+I+G+F | 1.00 | 1.00 | 0.00 |
|  |  | LG+I+G | 0.00 | 0.00 | 1.00 |
|  | Elision | LG+I+G+F | 1.00 | 1.00 | 1.00 |
|  |  | LG+I+G | 0.00 | 0.00 | 0.00 |
