# Supplementary material for: Evolution, gene expression profiling and 3D modeling of CSLD proteins in cotton
Source: BMC Plant Biol. 2017 Jul 10;17:119. doi: 10.1186/s12870-017-1063-x (PMC5504666; doi:10.1186/s12870-017-1063-x)
Supplement: Supplementary file 4 — Comparison of ML and Bayesian trees based on three alignments (Kalign, Mafft and Muscle) using Ktreedist. (DOCX 33 kb) [file 12870_2017_1063_MOESM4_ESM.docx]

| Trees | | K-score | Scale_factor | Symm_difference |
| --- | --- | --- | --- | --- |
| Reference | Comparison |  |  |  |
| Kalign_mrbayes^a^ | kalign_BS^b^ | 0.14 | 0.63 | 13 |
|  | kalign_SH^c^ | 0.13 | 0.76 | 11 |
| Mafft_mrbayes | mafft_BS | 0.15 | 0.63 | 12 |
|  | mafft_SH | 0.15 | 0.76 | 12 |
| Muscle_mrbayes | muscle_BS | 0.13 | 0.65 | 5 |
|  | muscle_SH | 0.13 | 0.77 | 5 |
| Elision_mrbayes | Elision _BS | 0.09 | 0.66 | 4 |
|  | Elision _SH | 0.09 | 0.80 | 4 |

**Additional file 4: Supplementary table S3. The comparison of ML and Bayesian trees based on three alignments (Kalign, Mafft and Muscle) using Ktreedist.**

^a^ We inferred the CSLD proteins phylogeny using by Bayesian methods.

^b^ We inferred the CSLD proteins phylogeny using by Maximum likelihood methods. Support values were estimated with bootstrap.

^c^  We inferred the CSLD proteins phylogeny using by Maximum likelihood methods. Support values were estimated with SH-like approximate likelihood.
