# Supplementary figures and images for: Evolution, gene expression profiling and 3D modeling of CSLD proteins in cotton
Source: BMC Plant Biol. 2017 Jul 10;17:119. doi: 10.1186/s12870-017-1063-x (PMC5504666; doi:10.1186/s12870-017-1063-x)

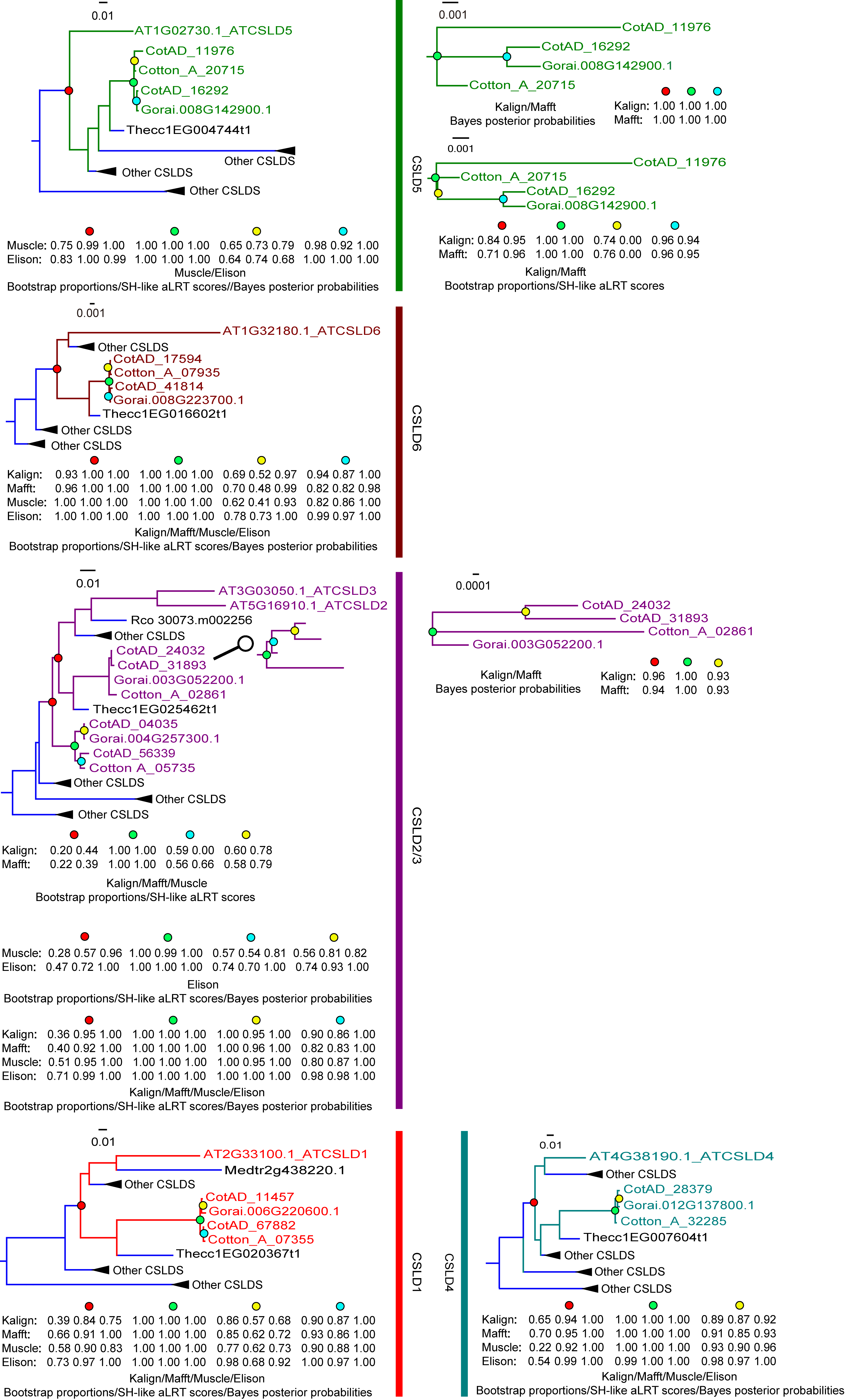

Supplement: Supplementary file 5 — The different topologies of cotton CSLD trees reconstructed from ML and Bayesian based on three alignments and the elision strategy. Support values are shown for A. thaliana-cotton and cotton CSLD nodes using different color circles as bootstrap proportions/SH-like aLRT scores/Bayesian posterior probabilities. The cotton CSLD protein clades are indicated by different colors. “Other CSLD” indicates the CSLD proteins from other plant species. (TIFF 2007 kb) [file 12870_2017_1063_MOESM5_ESM.tif]

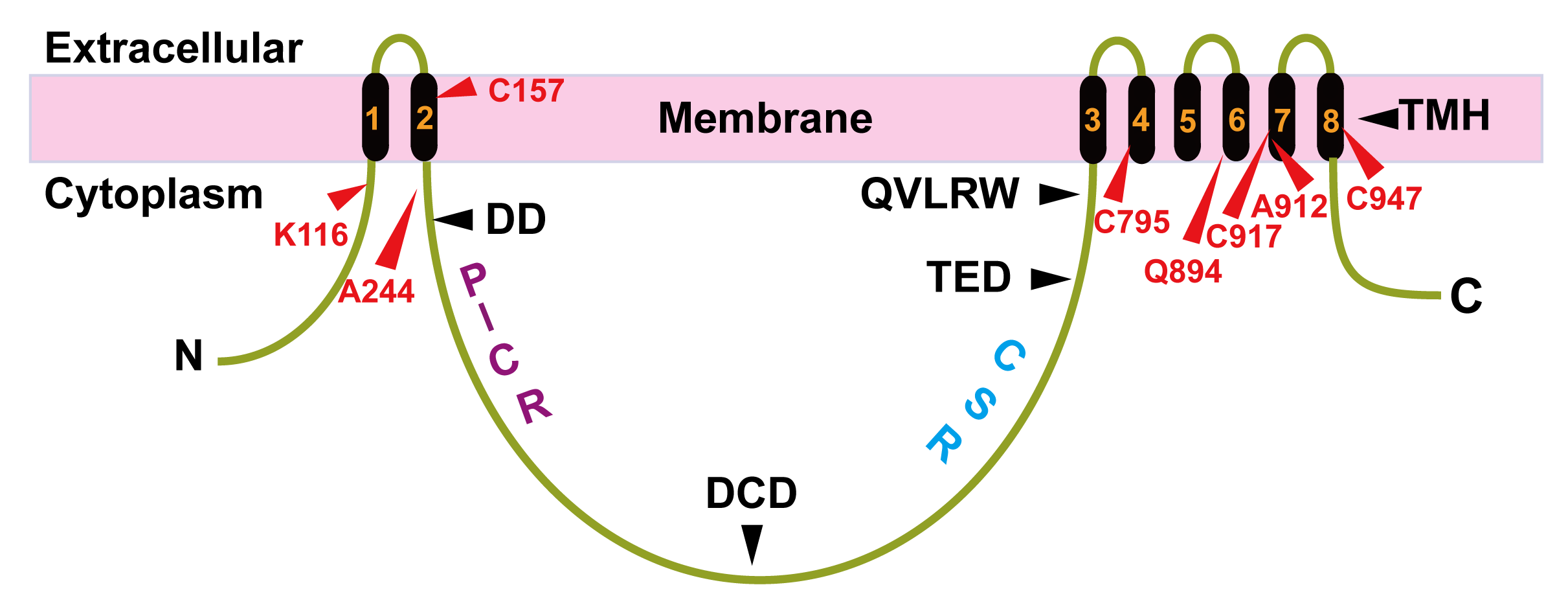

Supplement: Supplementary file 8 — Diagram of transmembrane helices (TMHs) and the cytosolic loop in GrCSLD1. The labels within the cytosolic loop and TMHs (1-8) show the approximate locations of the four conserved motifs (black), P-CR (purple), CSR (blue), and amino acid residues under positive selection (red). (TIFF 6688 kb) [file 12870_2017_1063_MOESM8_ESM.tif]

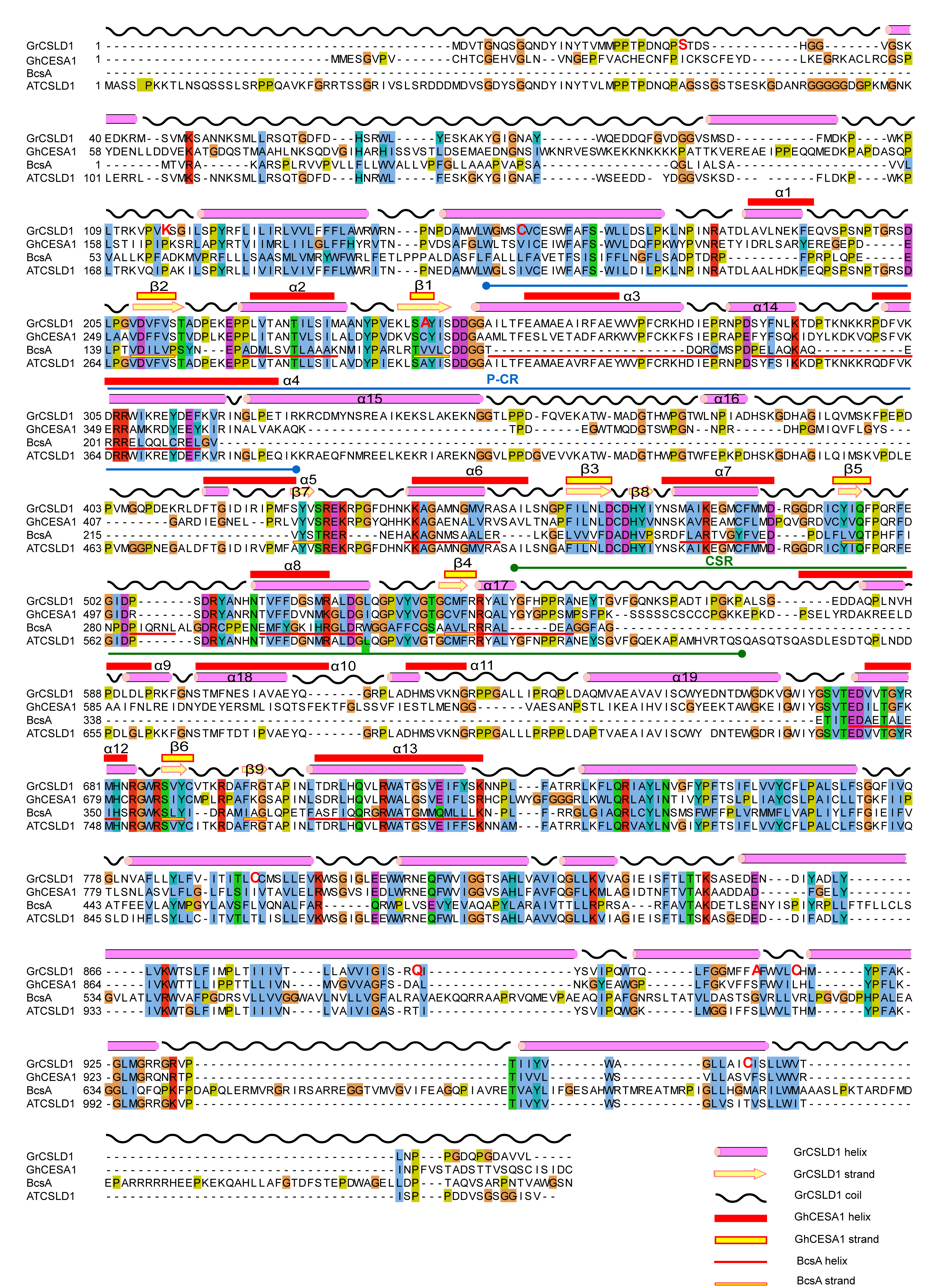

Supplement: Supplementary file 9 — Multiple sequence alignments of GrCSLD1, GhCESA1, BcsA and ATCSLD1. The secondary structure of GrCSLD1 was calculated using the DSS algorithm of PyMOL. The violet cylinders, yellow arrows, and black lines indicate the α-helices, β-strand and coil of GrCSLD1; the red rectangles and yellow rectangles indicate the α-helices and β-strand of GhCESA1, and the red lines and yellow lines indicate the α-helices and β-strand of BcsA. The plant-conserved region (P-CR) and class-specific region (CSR) are highlighted with blue and green lines. Large red letters indicate sites of episodic positive selection in GrCSLD1. (TIFF 4834 kb) [file 12870_2017_1063_MOESM9_ESM.tif]

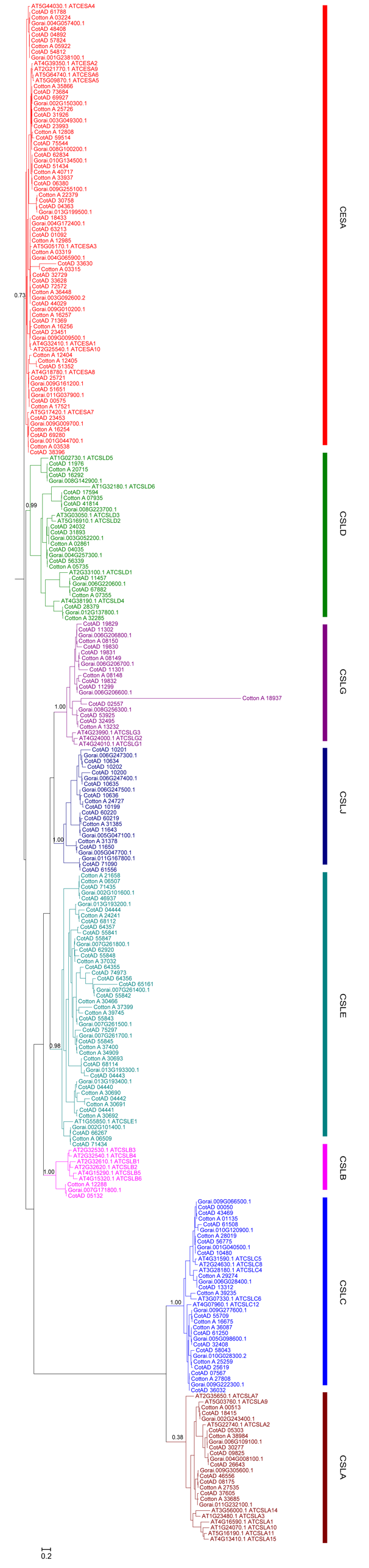

Supplement: Supplementary file 13 — Phylogenetic analysis of the CESA/CSL proteins in cotton and A. thaliana. The phylogenetic tree was inferred using maximum likelihood. Support values are shown for key nodes as bootstrap proportions. (TIFF 2921 kb) [file 12870_2017_1063_MOESM13_ESM.tif]
