# Supplementary material for: Evolution, gene expression profiling and 3D modeling of CSLD proteins in cotton
Source: BMC Plant Biol. 2017 Jul 10;17:119. doi: 10.1186/s12870-017-1063-x (PMC5504666; doi:10.1186/s12870-017-1063-x)
Supplement: Supplementary file 10 — Model validation scores of the full-length GrCSLD1 protein. (DOCX 22 kb) [file 12870_2017_1063_MOESM10_ESM.docx]

**Additional file 14: Supplementary table S15. Model validation scores of the full-length GrCSLD1 protein.**

| Name | Modeler  (DOPE score) | Verfity3D  (Averaged 3D-1D score>=0.2) | ProSA  (Z-score) |
| --- | --- | --- | --- |
| I-TASSER | -104312.14 | 58.16% | -7.65 |
| Phyre2 | -97004.16 | 60.54% | -4.05 |
| Robetta | -107587.45 | 80.79% | -9.02 |
